# Work Characteristics and Return to Work in Long-Term Sick-Listed Employees with Depressive Symptoms

**DOI:** 10.1007/s10926-017-9696-z

**Published:** 2017-01-28

**Authors:** Jenny J. J. M. Huijs, Lando L. J. Koppes, Toon W. Taris, Roland W. B. Blonk

**Affiliations:** 1 Behavioural and Societal Sciences, Sustainable Productivity and Employment, TNO, P.O. Box 3005, NL-2301 DA Leiden, The Netherlands; 2 NIVEL, Netherlands Institute for Health Services Research, Utrecht, The Netherlands; 3 Department of Social, Health and Organizational Psychology, Utrecht University, Utrecht, The Netherlands

**Keywords:** Return to work, Depression, Work characteristics, Self-efficacy, Sick leave

## Abstract

*Purpose* The present study investigated the relations between work characteristics, depressive symptoms and duration until full return to work (RTW) among long-term sick-listed employees. This knowledge may add to the development of effective interventions and prevention, especially since work characteristics can be subjected to interventions more easily than many disorder-related or personal factors. *Methods* this prospective cohort study with a two-year follow-up employs a sample of 883 Dutch employees who had been sick-listed for at least 13 weeks at baseline, who filled out three questionnaires: at 19 weeks, 1 and 2 years after the start of sick leave. The dependent measure was duration until full RTW. *Results* not working (partially) at baseline, low decision authority, high psychological demands, low supervisor support and low RTW self-efficacy were related to more depressive symptoms. The duration until full RTW was longer for employees with depressive symptoms. Low physical exertion, high RTW self-efficacy, working partially at baseline, being married or cohabiting, and young age were related to less time until full RTW. Other work characteristics appeared no independent predictors of RTW. *Conclusions* although the role of job demands and job resources in the RTW process is limited for long-term sick-listed employees with depressive symptoms, a few work characteristics are prognostic factors of full RTW. Focus on these elements in the selection or development of interventions may be helpful in preventing sickness absence, and in supporting long-term sick-listed employees towards full RTW.

## Introduction

Mental health problems have a high prevalence in the working population. Every year, one out of four adults in Europe suffers from psychological health issues [1]. Not only is the incidence of psychological disorders high; they often lead to long-term sickness absence and disability as well [2–4]. People who suffer from mental health problems are 30 to 50% less likely to be employed than those with other health problems or disabilities [4]. Several studies indicate that especially depressive symptoms adversely affect work status and duration until return to work (RTW) [5–7].

Depression is a common disorder, affecting over 350 million people worldwide and the leading cause for disability worldwide [8]. The lifetime prevalence of depression in general populations ranges from 10 to 15% [9]. In the working population, the 12-month prevalence rates of mood disorders varies between 4.2 and 6.4% [10, 11]. Depressive symptoms not only often coexist with physical disorders, particularly severe or chronic disorders such as cancer, musculoskeletal and cardiovascular diseases, but physical health problems can also cause depressive symptoms [12]. Depressive symptoms, especially when they culminate into a depressive disorder, are linked to several consequences, including lower labour market participation rates, stigmatization, lower socio-economic status, loss of a valuable source of social support, reduced quality of life and higher mortality rates [1, 9, 13]. Furthermore, employees might lose part of their income and tend to develop even more (severe) psychological symptoms [14]. Along with these individual consequences, the costs for society are also high because of productivity loss, medical consumption and disability benefits [1, 9]. In The Netherlands, depression is the largest contributor to the total number of sickness absence days with a mean duration of 200 days. The annual costs for society have been estimated at 1.8 billion Euros [15]. In Europe, the work-related costs due to psychological disorders are 2.5 times as high as those due to cardiovascular disorders [16].

The costs for individuals and society may decrease if employees on sick leave with depressive symptoms would RTW earlier. Unfortunately, at present there is no consistent evidence with respect to the factors that contribute to successful RTW for employees with depressive symptoms. A review on the factors that are related to work participation and work functioning among employees with depression showed that the literature mainly focuses on the onset of depression; research on factors that may promote or hinder RTW is relatively rare [17]. Even though there may be similarities, it is plausible that predictors of sick leave differ from the predictors of RTW [18]. Although Lagerveld et al. [17] identified 25 studies that investigated predictors of RTW for employees with depression, almost all of these focused on characteristics of the disorder. The duration of the current episode [19], severity of symptoms [e.g. 2, 20, 21], and co-morbidity are examples of these characteristics or disorder-related factors that lengthen the duration until RTW or are associated with work disability [e.g. 20, 22].

The role of work-related aspects for RTW for employees with depressive symptoms on sick leave, however, is hardly studied. A review [17] revealed only six studies that examined workplace factors [2, 19, 21–24]. There is some evidence that a previous low level of functioning at work is associated with increased work disability [19]. Also, contact between supervisor and other professionals besides the occupational physician is associated with a shorter duration until full RTW, but frequent contact with the supervisor during sick leave is related to a longer duration until full RTW [24]. The evidence for most of the examined workplace factors (like type of company, hours employed, type of occupation, position), however, is inconclusive or insufficient because of the study design (cross-sectional studies) or opposing findings. In addition, a Cochrane review on depressive disorders showed that there are only five studies on workplace interventions for employees with depressive disorders [25]. These five studies provide mixed results on sickness absence reduction.

Based on these results, the role of work characteristics in the RTW process of employees with depressive symptoms remains unclear. However, research in other populations indicates a relation between work characteristics and RTW or disability. High work demands were related to a lower chance of full RTW for employees on sick leave due to *general psychological complaints* [26]. In a population of employees on sick leave due to *low back pain*, high physical and psychological job demands and low supervisory support were related to 20% lower RTW rates, while high job control was related to 30% higher RTW rates [18]. Similar results were reported in three other studies: for employees in the public sector, low job control and high job insecurity were related to a 20–30% smaller chance of early rehabilitation [27], and high job strain (i.e. low job control and high job demands) was related to a 2.6 times higher odds for disability pension [28]. Furthermore, low job control and high work demands increased the risk of disability pension for construction workers [29]. Another study showed that employees on sick leave (for a maximum duration of 12 weeks at baseline) who received low coworker support had a longer duration until full RTW, whereas the duration until full RTW was shorter for those who experienced little supervisor support [30]. These studies suggest that work characteristics play an important role in the RTW process. Moreover, of the factors involved in the RTW process, employers can alter and subject work characteristics like job control, job demands and social support more easily to interventions and treatment than disorder-related or personal factors. Therefore, there is a need to evaluate work characteristics in relation to RTW in employees with depressive symptoms on sick leave. If the prognostic factors of RTW can be identified, a more adequate decision can be made in the selection or development of interventions.

Work related factors may affect mental health and functioning at work. A widely used theoretical model that describes the relations among these factors is the Job-Demand-Resources Model (JDR-Model) [31]. This model describes two connected processes, an erosion process and a motivational process. The erosion process describes how job demands (such as work pressure, psychological job demands, physical job demands) lead to a decrease in (mental) health through exhaustion. Job demands refer to “those physical, social or organizational aspects of the job that require sustained physical or mental effort and are therefore associated with certain physiological and psychological costs” [31]. In the motivational process, job resources (such as decision authority, social support, skill discretion) lead to an increase in (mental) health through engagement [32]. Job resources are defined as “those physical, psychological, social or organizational aspects of the job that may do any of the following: (a) be functional in achieving work goals; (b) reduce job demands at the associated physiological and psychological costs; (c) stimulate personal growth and development” [31]. Later, personal resources were added to the JDR-Model [32]. Personal resources refer to “an individuals’ sense of their ability to control and impact upon their environment successfully” [33]. Personal resources are aspects of the self that are generally linked to resiliency and refer to individuals’ sense of their ability to control and impact upon their environment successfully.

These personal resources (such as self-efficacy, self-esteem and optimism) have the same role in the motivational process as job resources. Previous research has shown that e.g. self-efficacy, self-esteem, optimism, hope and resilience are associated with engagement [32]. Specifically, as a personal resource the current study focuses on participants’ self-efficacy with regard to RTW [34].

The JDR-model describes how the balance between the two processes determines the health status of employees. Therefore, the model describes on the one hand the onset of complaints and disorders, and on the other hand the recovery from health issues [32]. The present study adds two concepts to the JDR-model. First, instead of common mental disorders, health issues are operationalized as depressive symptoms. Work factors and personal factors influence the recovery from common mental disorders like burnout, but the influence of work factors and personal factors on the recovery of more severe disorders such as depression is unknown [35]. Second, RTW is also entered in the model as an outcome. In the RTW process, job resources and personal resources will enhance RTW and job demands will hinder RTW. This prospective study among employees on long-term sick leave thus investigates the associations between job characteristics (job demands, job resources and personal resources), depressive symptoms and RTW. The specific cause of sick leave is not taken into account in this study. The following hypotheses are tested:

### Hypothesis 1a

Depressive symptoms are associated with lower job resources (skill discretion, decision authority, coworker support and supervisor support).

### Hypothesis 1b

 Depressive symptoms are associated with lower personal resources (RTW self-efficacy).

### Hypothesis 1c

Depressive symptoms are associated with higher job demands (psychological job demands, physical exertion and posture).

### Hypothesis 2

The duration until full RTW is longer for employees with depressive symptoms than for employees without depressive symptoms.

The hypotheses listed below are tested in the subgroup of employees with depressive symptoms.

### Hypothesis 3a

For employees with depressive symptoms, high job resources (skill discretion, decision authority, coworker support and supervisor support) shorten the duration until full RTW.

### Hypothesis 3b

For employees with depressive symptoms, high personal resources (RTW self-efficacy) shorten the duration until full RTW.

### Hypothesis 3c

For employees with depressive symptoms, high job demands (psychological job demands, physical exertion and posture) lengthen the duration until full RTW.

## Method

### Design and Procedure

The research population in this prospective cohort study consisted of a sample of Dutch employees on long-term sick leave. The participants of this study were recruited from the register of the Dutch Social Security Agency, that lists employees who are on sick leave for at least 13 weeks. Questionnaires were sent to each employee in the register that had a first day of sickness absence between May 19 and June 16 in 2007. The questionnaire was sent on the 5th of October 2007 to 10,118 employees, who were asked to fill out the questionnaire if they were still on (partial) sick leave. In total 2597 (26%) employees returned the questionnaire. Seventy-seven percent of these employees (*n* = 2000) met the inclusion criterion of being (partially) on sick leave. Non-response analysis showed that females and older employees returned the baseline questionnaire more often than others. On average, the participants had been on sick leave for 19 weeks when filling out the first questionnaire. In this first questionnaire, the participants were asked to sign up for the two follow-up questionnaires. The 1592 participants that signed up for the follow-up questionnaires were sent a second questionnaire (12–13 months after being sick-listed). Of these participants, 1090 people filled out this second questionnaire (response rate of 68%) and these respondents were sent a third questionnaire, 24–25 months after being sick listed. This final questionnaire was completed by 828 participants (response rate of 76%).

### Measures

The baseline questionnaire included information on socio-demographic characteristics, depressive symptoms, work characteristics, RTW self-efficacy and RTW. RTW was also measured 1 and 2 years after the start of sick leave. Eight single items measured *socio-demographic characteristics* (gender, age, level of education, ethnicity, marital status, presence of children in the household, type of contract, number of working hours according to contract before sick leave). *Depressive symptoms* were measured with the shortened self-report 10-item centre for epidemiologic studies depression (CES-D) scale [36]. An example item was: “I was bothered by things that usually don’t bother me”. Respondents were asked to describe how often they experienced each of these symptoms in the last week, with 0 = “Rarely or none of the time (less than 1 day)”, 1 = “Some or a little of the time (1–2 days)”, 3 = “Occasionally or a moderate amount of the time (3–4 days)”, and 4 = “Most or all of the time (5–7 days)”. The internal consistency in our study (Cronbach’s alpha) was 0.88. A sum score of 10 or greater was considered to signify depressive symptoms [36]. Although this study does not include information about a diagnosis or disorder, the original CES-D scale (cutoff score of 16) is validated with DSM-III criteria for clinical depression [36].

The *work characteristics* included seven concepts from the Job Content Questionnaire [37]. At baseline, employees were asked how they perceived their work before their sick leave. The response categories of all questions ranged from 0 (“completely disagree”) to 3 (“completely agree”). This study included three job demands. *Psychological job demands* were measured with four items (α = 0.78), including “My job requires working very fast”. Physical job demands were measured with two concepts: physical exertion and posture. *Physical exertion* was measured with three items, including “My job requires lots of physical effort” (α = 0.88). *Posture* consisted of two items with a reliability of 0.90. An example of an item was “I am often required to work for long periods with my body in physically awkward positions”.

Further, four job resources and one personal resource were measured. *Skill discretion* was tapped with five items, such as “My job requires me to be creative” (α = 0.70). *Decision authority* was measured with three items (α = 0.76), such as “I have a lot of say about what happens on my job”. Four items tapped *coworker support* (α = 0.82), such as “People I work with are friendly”. Four similar items tapped *supervisor support* (α = 0.88), including “My supervisor is concerned about the welfare of those under him”. As a personal resource, *RTW self-efficacy* was measured with 11 items [33], including: “If I resumed my work fully tomorrow I expect that I will be able to perform my tasks at work” (0 = “disagree entirely”, 5 = “agree entirely”, α = 0.92).

Finally, *RTW* was measured with the item “Are you working again at the moment?”. The answering categories were: “no, I am still sick” (0); “no, but I have been working in the mean time” (1); “yes, partially for … hours per week since …” (2); and “yes, fully since …” (3). RTW was operationalized as the length of time in calendar days from the start of sickness absence until full RTW, as reported by the participants in the questionnaires. Employees were considered to have returned to work fully if they indicated that they were working for at least the number of hours specified in their employment contract. Working on a therapeutic basis (i.e., with adjusted tasks or responsibilities) was not considered full RTW.

### Statistical Analysis

First, employees who filled out all variables were entered in our analysis. Second, all employees who reported they did not RTW because of other reasons than sick leave (e.g. because of retirement) at the second or third questionnaire, were removed from our analysis. In total, 883 employees met these criteria at the first follow-up (1 year after the start of sick leave) and 635 employees at the second follow-up (2 years after the start of sick leave). Differences at baseline between employees with and without depressive symptoms were tested with Pearson χ^2^ tests and *t*-tests. *T*-tests were also performed to study possible differences between these two groups in the duration until full RTW. To investigate which work characteristics were related to depressive symptoms at baseline, linear regression analyses were conducted. First, the relations among all work characteristics and RTW self-efficacy with depressive symptoms were assessed univariately at baseline. All variables that significantly associated with depressive symptoms were then tested in a multivariate linear regression analysis. In addition, this multivariate analysis was adjusted for the differences between those with and without depressive symptoms at baseline (gender, age, marital status) and for work status at baseline. Nonparametric Cox survival analysis was used to test the second and third hypotheses. The time lags used in our study were 1 and 2 years. To include participants that had not fully resumed work in our analysis, these individuals were given an artificial duration (censored observations) which was set at the number of days between start of sick leave and filling out the follow-up questionnaire. Survival analyses resulted in hazard ratios (HR) indicating a relative chance of full RTW. Thus, a HR larger than one signifies a higher chance of full RTW and therefore a shorter duration until full RTW.

To test hypothesis 3, the relations of all work characteristics and RTW self-efficacy at baseline with RTW were assessed univariately for both time lags. Again, all variables that were significantly associated with duration until full RTW were then tested in the multivariate models at both time lags. Multivariate analyses were adjusted for gender, age, marital status and work status at baseline.

## Results

### Baseline Characteristics

Participants at baseline were on average 46.4 years old, were more often female (56.3%), and had an average employment contract of 31.7 h per week (Table 1). Depressive symptoms (CES-D 10, cutoff score of 10 or greater) were reported at baseline by 438 (50%) of the 883 participants. On average, those with depressive symptoms were younger, more often female and more often without a partner. There are no differences between the two groups in duration of sick leave or work status at baseline. Almost 60% of the employees were (still) working partly at baseline, and for an average of 15.4 h per week. Employees with depressive symptoms worked the same number of hours at baseline as employees without depressive symptoms. 1 year after the start of sick leave, 320 people had not fully returned to work and 171 people were still on sick leave after 2 years (Table 2). Of the 540 respondents who were (partially) at work 2 years after the start of sick leave, almost 86% returned to their work at the same employer, 12.5% was employed by a different employer 2 years after the start of sick leave. At both follow-ups, employees with depressive symptoms were less likely to have returned to work fully, as compared to employees without depressive symptoms at baseline. In addition, the duration until full RTW was longer for employees with depressive symptoms. 1 year after the start of sickness absence the difference was approximately 30 calendar days in favor of those without depressive symptoms at baseline, at the second follow-up the difference was over 50 days.

**Table 1:** Baseline characteristics of the participants

		Depressive symptoms		
		No	Yes	Total
	N	445	438	883
	%	50%	50%	100%
Gender	Male	48.3%▴▴	39.0%▾▾	43.7%
	Female	51.7%▾▾	61.0%▴▴	56.3%
Age	Mean	47.2▴▴	45.5▾▾	46.4
	Standard deviation	8.9	9.4	9.2
Marital status	No partner	16.9%▾▾▾	26.3%▴▴▴	21.5%
	Married or cohabiting	83.1%▴▴▴	73.7%▾▾▾	78.5%
Working hours according to contract	Mean	31.9	31.4	31.7
	Standard deviation	9.5	8.5	9.0
Children living at home	No	45.8%	50.9%	48.4%
	Yes	54.2%	49.1%	51.6%
Education	Lower education	45.0%	39.9%	42.5%
	Intermediate or higher education	55.0%	60.1%	57.5%
Ethnicity	Native	89.9%	89.5%	89.7%
	Immigrant	10.1%	10.5%	10.3%
Contract type	Permanent position	95.1%	94.7%	94.9%
	Fixed term contract	4.9%	5.3%	5.1%
Working hours	≤32 h (part-time)	43.1%	47.2%	45.1%
	>32 h (full-time)	56.9%	52.8%	54.9%
Organization size	0–49 employees	24.5%	26.5%	25.5%
	50–499 employees	34.9%	32.7%	33.8%
	500 or more employees	40.6%	40.8%	40.7%
Depressive symptoms	Mean	4.86▾▾▾	15.9▴▴▴	10.4
	Standard deviation	2.63	4.67	6.71
Duration of sick leave	Mean	18.7	18.8	18.8
	Standard deviation	1.53	1.65	1.59
Working status at Baseline	Fully sick listed	38.7%	44.7%	41.7%
	Partial RTW	61.3%	55.3%	58.3%
Hours working at baseline	Mean	15.7 (N = 258)	15.1 (N = 231)	15.4 (N = 489)
	Standard deviation	8.6	8.1	8.4

▴▴p < 0.01, ▴▴▴p < 0.001 (and ▾) significantly high (low) percentages and/or means

**Table 2:** RTW characteristics of the participants

		Depressive symptoms		
		No	Yes	Total
Days until full RTW as measured at 1 year after the start of svick leave	Mean	279.1▾▾▾	310.7▴▴▴	294.7
	SD	105.4	93.0	100.6
	N	445	438	883
Working status at 1 year after the start of sick leave	Fully sick listed	14.8%▾▾	23.1%▴▴	18.9%
	Partial RTW	16.9%	17.8%	17.3%
	Full RTW	68.3%▴▴	59.1%▾▾	63.8%
	N	445	438	883
Days until full RTW as measured at 2 years after the start of sick leave	Mean	430.5▾▾	483.9▴▴	456.5
	SD	235.3	238.9	238.4
	N	326	309	635
Working status at 2 years after the start of sick leave	Fully sick listed	10.1%▾▾▾	20.1%▴▴▴	15.0%
	Partial RTW	12.3%	11.7%	12.0%
	Full RTW	77.6%▴▴	68.3%▾▾	73.1%
	N	326	309	635

▴▴p < 0.01, ▴▴▴p < 0.001 (and ▾) significantly high (low) percentages and/or means

### Associations of Work Characteristics and RTW Self-Efficacy with Depressive Symptoms at Baseline

Univariate analyses showed that all work characteristics (except physical exertion) and RTW self-efficacy were associated with depressive symptoms (Table 3). In the multivariate model, several characteristics remained significantly associated with depressive symptoms. Employees who were married or cohabiting were less likely to have depressive symptoms. Further, employees who worked partially at baseline, who had a higher level of decision authority, a lower level of psychological job demands or who had a higher level of RTW self-efficacy, had fewer depressive symptoms at baseline. Furthermore, female employees and employees who experienced less social support from their supervisor were slightly more likely to have depressive symptoms. Hence, the results are in line with hypothesis 1a for the job resources decision authority and supervisor support. Furthermore, the results are in line with hypothesis 1b: high personal resources are associated with a lower chance for depressive symptoms. Hypothesis 1c was in line with the results for psychological job demands, but not for the job demands physical exertion and posture.

**Table 3:** Correlates of depressive symptoms at baseline

	Depressive symptoms	
	Univariate	Multivariate
	β	β
Gender (ref = male)	0.09***	0.05*
Age	−0.09***	−0.03
Marital status (ref = no partner)	−0.16***	−0.11***
Working partially at baseline (ref = no)	−0.14***	−0.06**
Skill discretion	−0.09***	0.01
Decision authority	−0.20***	−0.08**
Psychological job demands	0.23***	0.10***
Physical exertion	−0.03	–
Posture	0.07**	0.01
Coworker support	−0.15***	−0.04
Supervisor support	−0.24***	−0.06*
RTW self-efficacy	−0.50***	−0.42***
N	883	883
Adjusted R-square		0.29

*p < 0.10, **p < 0.05, ***p < 0.01

### Associations of Depressive Symptoms with RTW Within 1 Year and 2 Years After the Start of Sick Leave

Employees with depressive symptoms had a longer duration until full RTW (HR = 0.97 at both follow-ups) (Table 4). Furthermore, working status at baseline predicted full RTW. For employees who worked partially at baseline, the duration until full RTW was shorter (HR = 2.53 at 1 year and HR = 1.85 at 2 years after the start of sick leave). 2 years after the start of sick leave, higher age was related to a longer duration until full RTW (HR = 0.98). Therefore, the results are in line with hypothesis 2.

**Table 4:** Multivariate associations of depressive symptoms with the duration until full RTW at 1 year and 2 years after the start of sick leave

	1 year after start of sick leave (N = 883)		2 years after start of sick leave (N = 635)	
	HR (95% CI)	*P*	HR (95% CI)	*P*
Gender (ref = male)	0.93 (0.78–1.11)	0.43	1.08 (0.88–1.31)	0.47
Age	0.99 (0.98–1.00)	0.16	0.98 (0.97–0.99)	<0.01
Marital status (ref = no partner)	0.95 (0.77–1.18)	0.66	1.08 (0.85–1.38)	0.52
Working partially at baseline (ref = no)	2.53 (2.11–3.04)	<0.01	1.85 (1.52–2.24)	<0.01
Depressive symptoms	0.97 (0.96–0.98)	<0.01	0.97 (0.96–0.99)	<0.01

HR (hazard ratio) of >1 indicates earlier RTW

### Associations of Work Characteristics and RTW Self-Efficacy with RTW for People with Depressive Symptoms Within 1 Year and 2 Years After the Start of Sick Leave

Univariate analyses showed that psychological job demands, physical exertion, posture, skill discretion and RTW self-efficacy significantly predicted duration until full RTW within 1 or 2 years after the start of sick leave for people with depressive symptoms. However, in the multivariate models only RTW self-efficacy and (to a smaller extent) physical exertion^1^ remained significant predictors of full RTW (Table 5). Hence, the results are only in line with hypothesis 3b. A higher level of RTW self-efficacy at baseline, was related to a shorter duration until full RTW at both follow-ups (HR = 1.19 and HR = 1.20). 1 year after the start of sick leave, a higher level of physical exertion was related to a slightly longer duration until full RTW (HR = 0.84). In addition, working status at baseline, marital status and age predicted full RTW. For employees who worked partially at baseline, the duration until full RTW was shorter (HR = 2.80 at first follow-up and HR = 1.80 at second follow-up). Having a partner at baseline (HR = 1.56) was related to a shorter duration and higher age at baseline (HR = 0.97) to a longer duration until full RTW 2 years after the start of sick leave.

**Table 5:** The relation of work characteristics with duration until full RTW for people with depressive symptoms at 1 and 2 years after the start of sick leave

	1 year after start of sick leave				2 years after start of sick leave			
	Univariate		Multivariate		Univariate		Multivariate	
	N_min_ = 456 N_max_ = 464		N = 438		N_min_ = 327 N_max_ = 333		N = 309	
	HR (95% CI)	*P*	HR (95% CI)	*P*	HR (95% CI)	*P*	HR (95% CI)	*P*
Gender (ref = male)	0.89 (0.69–1.13)	0.33	0.86 (0.66–1.11)	0.25	1.01 (0.76–1.32)	0.97	0.99 (0.73–1.33)	0.94
Age	1.00 (0.99–1.01)	0.70	0.99 (0.98–1.00)	0.15	0.98 (0.97–0.99)	<0.01	0.97 (0.96–0.99)	<0.01
Marital status (ref = no partner)	1.19 (0.90–1.57)	0.22	1.19 (0.89–1.59)	0.24	1.43 (1.04–1.97)	0.03	1.56 (1.11–2.19)	0.01
Working partially at baseline (ref = no)	3.04 (2.34–3.95)	<0.01	2.80 (2.13–3.68)	<0.01	1.90 (1.44–2.50)	<0.01	1.80 (1.34–2.41)	<0.01
Psychological job demands	1.12 (0.90–1.38)	0.31	1.12 (0.89–1.40)	0.34	1.30 (1.03–1.65)	0.03	1.24 (0.95–1.62)	0.12
Physical exertion	0.86 (0.75–0.99)	0.04	0.84 (0.68–1.03)	0.09	0.88 (0.75–1.03)	0.11	0.84 (0.65–1.09)	0.19
Posture	0.85 (0.73–0.98)	0.03	1.06 (0.86–1.31)	0.60	0.85 (0.72–1.01)	0.07	1.05 (0.81–1.38)	0.70
Skill discretion	1.19 (0.93–1.53)	0.16	0.95 (0.73–1.24)	0.72	1.50 (1.14–1.99)	<0.01	1.13 (0.82–1.57)	0.46
RTW self-efficacy	1.26 (1.13–1.39)	<0.01	1.19 (1.06–1.33)	<0.01	1.19 (1.07–1.33)	<0.01	1.20 (1.06–1.35)	<0.01

HR (hazard ratio) of >1 indicates earlier RTW

*N*_*min*_ lowest number of respondents in the analyses

*N*_*max*_ highest number of respondents in the analyses

## Discussion

The present study investigated the relation between depressive symptoms, work characteristics and duration until full RTW among employees on long-term sickness absence. Firstly, the present study showed several relations between work characteristics and depressive symptoms: a higher level of decision authority, a lower level of psychological demands, more social support from the supervisor and a higher level of RTW self-efficacy were associated with a lower chance of reporting depressive symptoms at baseline. This is in line with earlier studies. For instance, Plaisier et al. [38] showed that the risk of depression increased with a higher level of psychological demands or a lower level of daily emotional support. High levels of (psychological) demands, low levels of decision latitude or job control, and low levels of social support at work were predictors of depression or other psychiatric disorders [39–41].

Secondly, depressive symptoms were strong predictors of the duration until full RTW. When we compared the employees with depressive symptoms to those without such symptoms, employees with depressive symptoms needed 30–50 days more days to full RTW at both follow-ups. This negative association between depressive symptoms and RTW is in line with previous research [e.g. 5–7].

Finally, although the hypothesis was that high levels of job and personal resources would shorten, and high levels of job demands would lengthen the time until full RTW for employees with depressive symptoms, this study showed only associations of RTW self-efficacy (a personal resource) and physical exertion (a job demand) with RTW. As expected, the higher the level of RTW self-efficacy at baseline, the earlier employees returned to work fully. This association of self-efficacy was reported in other studies as well [42, 43]. A lower level of RTW self-efficacy can lead to less confidence of employees that they will succeed in the work environment, leading them to avoid this setting. Consequently, these employees will need more time to fully RTW. Conversely, employees with a high level of RTW self-efficacy will have more confidence in their ability to face the challenges in the workplace and therefore their time to fully RTW will be shorter. A study by Nieuwenhuijsen, Noordik, Van Dijk and Van der Klink [44] shows that lower levels of fatigue, depressive symptoms, work pace and workload are associated with higher levels of RTW self-efficacy. Thus, although the present study shows that the role of job demands and job resources in the RTW process is limited, work characteristics may influence RTW self-efficacy.

1 year after the start of sick leave, a higher level of physical exertion was related to a slightly longer duration until full RTW, which is consistent with the findings of Schultz et al. [45]. In their study RTW was related to lower physical work demands. High levels of physical work demands are not only related to lower RTW rates, but also to long-term consequences as work disability [18, 29].

In addition to RTW self-efficacy and physical exertion, partial RTW at baseline was related to a shorter duration until full RTW. Partial RTW can be viewed as a type of gradual exposure to the work situation and may provide successful work experiences that challenge the dysfunctional beliefs an employee might have about work and RTW [46]. The modification of dysfunctional beliefs is one of the basic mechanisms that explain the effectiveness of (gradual) exposure [47, 48]. The findings of the present study are in line with research that showed that graded work exposure enhances full RTW [e.g. 46, 49].

Older employees and employees without a partner had a longer duration until full RTW. These results are similar to the findings in other studies [e.g. 17, 50]. One explanation for the association of age is that older employees may need more time to recover from their depressive symptoms and therefore have a longer duration until full RTW. Another possibility is that older employees more often aim at retirement or pre-pension instead of RTW. However, to our knowledge no study has investigated this.

The present study found only few associations between work characteristics and RTW for employees with depressive symptoms. This is not uncommon. Studies on workers with low back pain or common mental disorders also found few direct associations between work characteristics and RTW or disability [6, 45]. One explanation for these results draws on the populations that are used in these studies. The duration of sick leave at start of the study differs enormously across these studies. Some studies used an inclusion criterion of at least 1 day of sick leave, while in other studies employees had to report sick for at least 4 weeks. Studies that only include employees on long-term sick leave use a wide variation in the definition of long-term sick leave. Most studies excluded employees on sick leave for 12 weeks or more at baseline [e.g. 7, 30, 43]. The present study, however, included only people who were on sick leave for *at least* 13 weeks. Work characteristics may play a more important role in the RTW process for people on short-term sick leave. The employees’ perceptions of job demands as work load and emotional demands, but also job resources as decision latitude, may influence the decision to RTW. Nevertheless, the present study finds no independent associations for job demands and job resource with RTW. If people are already for more than 13 weeks on sick leave, the RTW process may be more multilayered with a more diverse range of factors that play a role in work resumption. Future research could address this issue and study similar populations longitudinally and investigate several predictors (work characteristics, disorder characteristics, individual factors and social and economic aspects) simultaneously.

### Strengths and Limitations

The present study is among the first to examine the associations of work characteristics with RTW for sick-listed employees with depressive symptoms. All the participants in this study were absent for at least 13 weeks. The average sick leave duration when returning the first questionnaire was 19 weeks. In addition, the follow-up period was 2 years. Such populations have as yet not often been studied. Most studies about employees on long-term sick leave apply an exclusion criterion of maximum sick leave duration of 12 weeks at baseline and a 1-year follow-up [e.g. 7, 30, 43]. The present study is among the very few studies that combine a follow-up period of 2 years with a sick leave period at baseline of more than 12 weeks.

Apart from these strengths, three main limitations of the present study must be noted. First, the initial response rate was only 26%. Given the heterogeneous sample of long-term sick listed employees and contamination of the national registration, estimated at around 40% and mostly due to lacking resumption notifications, a higher response rate was not expected. Further, although nonresponse analysis revealed some differences between responders and nonresponders, overall these differences were small. Note that a low initial response rate is not uncommon in this area. Baruch and Holtom [51] argued that the average response rate of studies in organizational research is often low because of difficulties reaching the target population and the reluctance of the people to respond. However, the response rate of the follow-ups of our study is high (i.e. 68 and 76%), especially when considering the long follow-up period of 20 months. Furthermore, the overall response of almost 800 respondents who returned all three questionnaires is high in comparison with other similar studies [e.g. 5, 24, 44].

Second, it should be noted that although RTW was measured prospectively, the data on the work characteristics were gathered retrospectively. The retrospective measurement may have led to a recall bias. Moreover, almost 60% of the participants were partially at work at baseline. Their present work experiences may have affected their opinion on the characteristics of their work prior to the start of their sick leave. Similarly, the response of those employees who were still fully on sick leave may have been influenced by their experiences during sick leave, because the baseline measurement was 19 weeks after the start of sick leave.

Third, unfortunately, this study only measured depressive symptoms and not (clinical) depression (e.g. diagnosis, disorder or sick leave origin). Other research has shown that the cutoff score that is used in the original 20-item CES-D scale (cutoff score of 16) is validated with DSM-III criteria for clinical depression [36]. In addition, the shortened CES-D has good predictive accuracy when compared to the 20-item version [36]. Therefore, it is hypothesized that the participants in this study who score above the cutoff score of the CES-D (10 or higher) have severe depressive symptoms that are at least close to a clinical depression. Depressive symptoms were not necessarily the cause of sick leave in our sample. Employees could be on sick leave due to physical and/or psychological disorders.

## Conclusion

The present study investigated the role of work characteristics in the RTW process of Dutch employees with depressive symptoms and long-term sick leave. This study shows that work characteristics are associated with depressive symptoms. Employees who work partially at baseline, have a higher level of decision authority, a lower level of psychological demands or who have a higher level of RTW self-efficacy, were less likely to report depressive symptoms at baseline. Furthermore, those who experience less social support from their supervisor are slightly more likely to have depressive symptoms. In addition, the duration until full RTW is longer for employees with depressive symptoms. Only few associations are found between work characteristics and RTW. Physical exertion, RTW self-efficacy, work status at baseline, marital status and age are significant independent predictors of full RTW. This study suggests that work characteristics may influence depressive symptoms, but that their role in the RTW process is limited for employees with depressive symptoms on long-term sick leave. Knowledge of prognostic factors of RTW for long-term sick-listed employees with depressive symptoms is still fragmented and limited. As RTW may be helpful in the recovery of depressive symptoms, a better insight in factors that facilitate RTW can lead to more adequate choices in the selection or development of interventions and can also be used to prevent long-term sickness absence.

## Footnotes

1 The HR of physical exertion is almost constant in the univariate and multivariate models. But because of the increase of variance in the multivariate model the significance drops to 0.09 (and 0.19).
